# Reversible off and on switching of prion infectivity via removing and reinstalling prion sialylation

**DOI:** 10.1038/srep33119

**Published:** 2016-09-09

**Authors:** Elizaveta Katorcha, Martin L. Daus, Nuria Gonzalez-Montalban, Natallia Makarava, Peter Lasch, Michael Beekes, Ilia V. Baskakov

**Affiliations:** 1 Center for Biomedical Engineering and Technology, University of Maryland School of Medicine, Baltimore, Maryland, 21201 United States of America; 2 Department of Anatomy and Neurobiology, University of Maryland School of Medicine, Baltimore, Maryland, 21201 United States of America; 3 Centre for Biological Threats and Special Pathogens, Robert Koch-Institute, 13353 Berlin, Germany

## Abstract

The innate immune system provides the first line of defense against pathogens. To recognize pathogens, this system detects a number of molecular features that discriminate pathogens from host cells, including terminal sialylation of cell surface glycans. Mammalian cell surfaces, but generally not microbial cell surfaces, have sialylated glycans. Prions or PrP^Sc^ are proteinaceous pathogens that lack coding nucleic acids but do possess sialylated glycans. We proposed that sialylation of PrP^Sc^ is essential for evading innate immunity and infecting a host. In this study, the sialylation status of PrP^Sc^ was reduced by replicating PrP^Sc^ in serial Protein Misfolding Cyclic Amplification using sialidase-treated PrP^C^ substrate and then restored to original levels by replication using non-treated substrate. Upon intracerebral administration, all animals that received PrP^Sc^ with original or restored sialylation levels were infected, whereas none of the animals that received PrP^Sc^ with reduced sialylation were infected. Moreover, brains and spleens of animals from the latter group were completely cleared of prions. The current work established that the ability of prions to infect the host via intracerebral administration depends on PrP^Sc^ sialylation status. Remarkably, PrP^Sc^ infectivity could be switched off and on in a reversible manner by first removing and then restoring PrP^Sc^ sialylation.

Prion diseases are a family of transmissible neurodegenerative maladies caused by misfolding and aggregation of a cellular sialoglycoprotein called the prion protein or PrP^C^ into a conformationally altered, self-replicating, disease-associated state referred to as PrP^Sc ^1. While prions or PrP^Sc^ are not conventional pathogens and lack coding nucleic acids, they can be transmitted effectively via natural routes and characterized by high infectivity titers akin to conventional pathogens^2^,^3^,^4^.

The first line of defense against microbial pathogens in mammals is provided by the innate immune system. To recognize potential pathogens, the innate immune system senses a number of molecular features that discriminate pathogens from host cells. Among them is sialylation, a terminal modification of glycans that is found on all types of mammalian cells, but is absent on most microbial pathogens^5^,^6^. To the cells of the innate immune system, sialylated glycans act as part of a “self-associated molecular pattern”, whereas asialoglycans with exposed galactose residues act as part of a “pathogen-associated molecular pattern”^5^,^6^. Exposed galactose on carbohydrates is believed to serve as “eat me” signal for professional and non-professional microphages including microglia^7^,^8^. PrP^C^ is posttranslationally modified with one or two N-linked glycans, in which terminal sialic acid residues are attached to galactose residues via α2–3 or α2–6 linkages^9^,^10^,^11^. Upon conversion into PrP^Sc^, the N-linked glycans of PrP^C^ are carried over giving rise to sialylated PrP^Sc^ 12,13. Previously, we proposed that due to sialylation, PrP^Sc^ is not recognized by the innate immune system as a potential pathogen^14^,^15^. The current study was designed to test the hypothesis that sialylation represents a key molecular feature that controls infectivity of PrP^Sc^, even when it is administered directly into brain, avoiding any encounter with the innate immune system in the periphery. In particular, we asked whether the infectivity of PrP^Sc^ could be switched off and on in a reversible manner by removing and reinstalling sialic acid residues.

In the CNS microglia, astrocytes, endothelial cells and pericytes perform the functions that are fulfilled by the innate immune system in the periphery (reviewed in ref. 16). In the periphery, commensal bacteria that colonize surfaces early in life act in sync with the host innate immune system, helping to prevent invasion of pathogens^17^,^18^. In fact, commensal bacteria are believed to calibrate the activation threshold of the innate immune system, ensuring a balanced immune response against pathogens but not against self^17^,^18^. Because the CNS lacks direct exposure to commensal bacteria, it is not clear whether the defense mechanisms of the innate immune system that evolved to rely on sialylation or its lack thereof are also applicable to the CNS.

To test the above hypothesis, the current study used SSLOW, a prion strain of synthetic origin^19^. Just like other prion strains, SSLOW is transmissible, displays a high infectivity titer and causes neuronal loss, gliosis and spongiform vacuolation^4^,^20^,^21^. It is deposited in the form of large plaques and small synaptic and perineuronal deposits^19^,^20^,^21^. Previously, SSLOW deposits were found in association with neuronal and glial cells^19^,^20^,^21^. However, in contrast to the vast majority of rodent strains, including 263 K or Hyper, SSLOW exhibits a long incubation time to disease and slow progression of the clinical disease^20^, which is more typical of human and large animal Transmissible Spongiform Encephalopathies than those of rodents. In addition, SSLOW-infected animals showed substantial cerebral amyloid angiopathy^19^,^21^, a pathogenic feature often observed in a subset of individuals with Alzheimer’s disease. SSLOW replicates very efficiently in Protein Misfolding Cyclic Amplification with beads (PMCAb)^22^,^23^, making the production of SSLOW with modified sialylation status *in vitro* or detection of miniscule amounts of SSLOW PrP^Sc^ in animal tissues feasible.

To modify the sialylation status of SSLOW PrP^Sc^, the current work employed serial PMCAb conducted with normal brain homogenate (NBH) in which the sialylation status of PrP^C^ was first reduced using sialidase treatment and then restored to the original level by propagating using non-treated NBH. We found that the ability of PrP^Sc^ to infect animals via intracerebral (IC) administration depended on the PrP^Sc^ sialylation status. Remarkably, we showed that the infectivity of SSLOW PrP^Sc^ could be switched off and on in a reversible manner by removing and then restoring PrP^Sc^ sialylation, respectively. This study suggests that controlling sialylation status of PrP^Sc^ might open new opportunities for therapeutic intervention against prion diseases.

## Results

**Figure 1:**
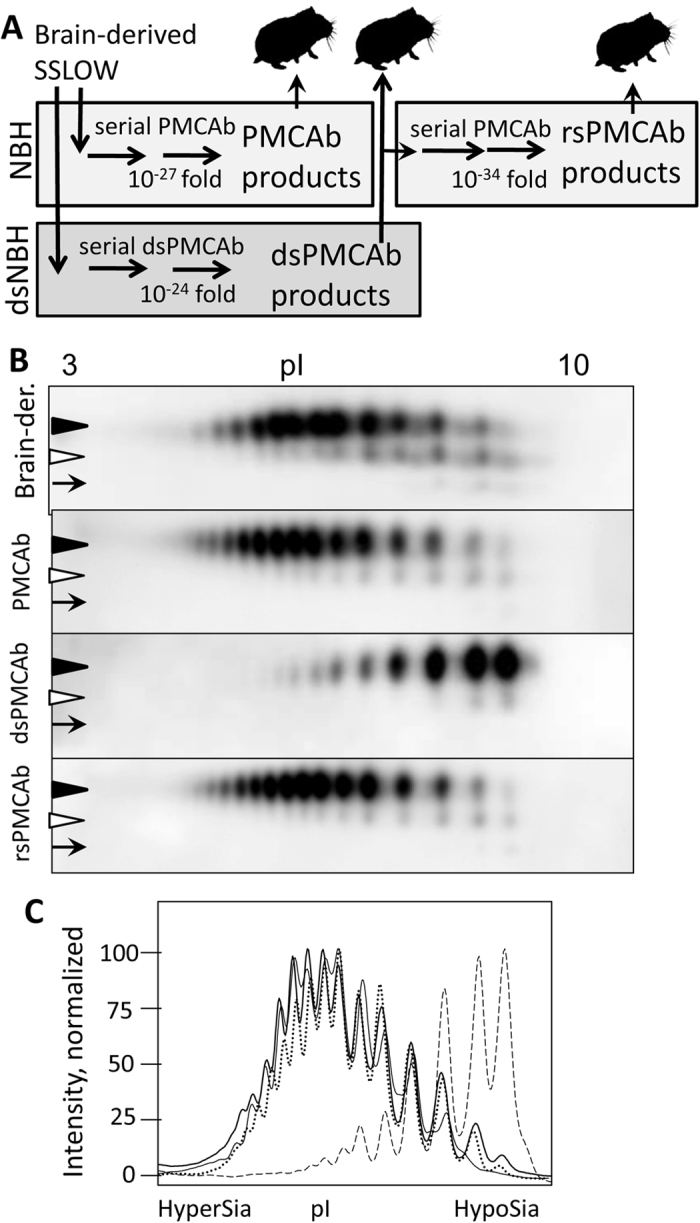
Experimental design and 2D analysis of sialylation status. (**A**) Experimental design illustrating generation of desialylated and re-sialylated SSLOW material. To produce desialylated SSLOW, serial dsPMCAb reactions were seeded with brain-derived SSLOW and conducted using sialidase-treated NBH to a final dilution of SSLOW brain material of 10^−24^-fold. To produce re-sialylated SSLOW, serial rsPMCAb was seeded with dsPMCAb products and conducted using non-treated NBH to a final dilution of original SSLOW brain material 10^−34^-fold. To produce reference PMCAb-derived material, serial PMCAb reaction was seeded with brain-derived SSLOW and conducted using non-treated NBH to a final dilution of SSLOW brain material 10^−27^-fold. Animals were inoculated IC with 10-fold diluted PMCAb, dsPMCAb- or rsPMCAb-derived material. (**B**) 2D Western blot analysis of SSLOW brain-, PMCAb-, dsPMCAb- and rsPMCAb-derived material. Black and white triangles mark diglycosylated and monoglycosylated glycoforms, respectively, whereas arrows mark the unglycosylated form. All blots were stained with 3F4 antibody. (**C**) Sialylation profiles of diglycosylated isoforms of SSLOW brain- (solid thin line), PMCAb- (solid bold line), dsPMCAb- (dashed line) and rsPMCAb-derived material (dotted line). Profiles were built as described in Materials and Methods using results of 2D Western blots.

Previous studies established that within brain-derived PrP^Sc^, PrP molecules are highly heterogeneous with respect to sialylation status of their N-linked glycans ranging from hyposialylated to hypersialylated^14^,^24^,^25^. To produce PrP^Sc^ with reduced sialylation status, serial PMCAb reactions were seeded with brain-derived SSLOW PrP^Sc^, and sialidase-treated normal brain homogenate (dsNBH) was used as a source of PrP^C^ (Fig. 1A). The resulting material produced in dsNBH will be referred to as dsPMCAb-derived SSLOW. Direct treatment of brain-derived PrP^Sc^ with sialidase was not effective, presumably due to the highly aggregated nature of PrP^Sc^ and limited access of sialic acid residues to sialidases. To produce a reference PMCAb-derived SSLOW, serial PMCAb reactions were seeded with brain-derived SSLOW PrP^Sc^, and the reactions were conducted using NBH as a substrate in the absence of sialidase treatment. Multiple serial rounds were conducted for both dsPMCAb and PMCAb reactions to make sure that the final dilutions of the original SSLOW seeds in dsPMCAb or PMCAb products were well beyond the limiting dilution (Fig. 1A). To restore the sialylation status of dsPMCAb-derived SSLOW, serial PMCAb reactions were seeded with dsPMCAb-derived SSLOW and subjected to replication in NBH (Fig. 1A). The resulting material will be referred to as resialylated or rsPMCAb-derived SSLOW.

The sialylation status of the original brain-derived SSLOW, as well as PMCAb-, dsPMCA- and rsPMCA-derived SSLOW, was assessed using two-dimensional Western blot (2D). Prior to 2D, all samples were treated with PK, and then the PrP^Sc^ aggregates were denatured into PrP monomers. The sialylation status of individual PrP molecules is reflected by their positions on the horizontal dimension of 2D, where heavily sialylated PrPs run toward acidic pHs, whereas weakly sialylated ones run toward basic pHs. Brain-, PMCAb- and rsPMCA-derived SSLOW showed a very similar distribution of charge isoforms, confirming the very similar sialylation status of PrP^Sc^ in these three materials (Fig. 1B,C). All three samples showed a broad sialylation pattern with considerable predominance of highly sialylated isoforms. Notably, the sialylation of rsPMCA-derived SSLOW was restored to the levels characteristic of brain- or PMCAb-derived SSLOW. As expected, the sialylation level of dsPMCA-derived SSLOW was substantially lower in comparison to those of brain-, PMCAb- or rsPMCA-derived SSLOW (Fig. 1B,C).

**Figure 2:**
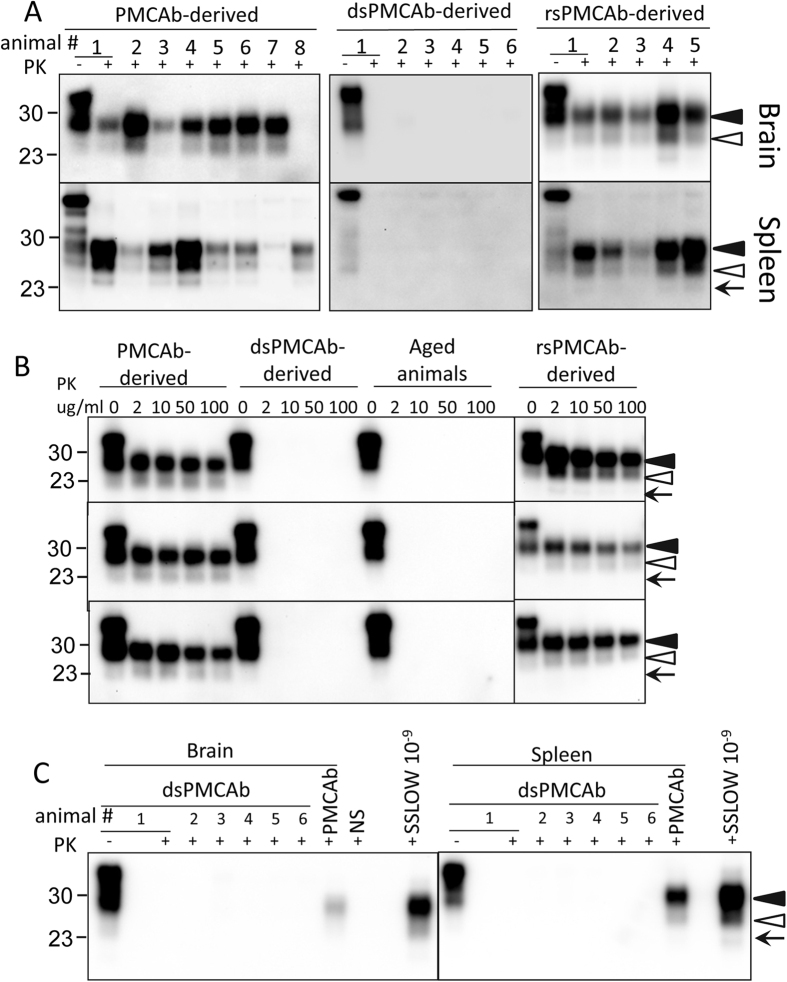
Analysis of brains and spleens from animals inoculated with SSLOW PMCAb-, dsPMCAb- or rsPMCAb-derived materials. Syrian hamsters were inoculated IC with 10-fold diluted PMCAb-, dsPMCAb- or rsPMCAb-derived material. (**A**) Brain or spleen homogenates were treated with PK and analyzed by Western blot. (**B**) Western blots of brain homogenates from animals inoculated with PMCAb-, dsPMCAb- or rsPMCAb-derived material or non-inoculated animals 600–670 days old. Brain homogenates were treated with increasing concentrations of PK as indicated. PK-digestion profiles for three independent animals are shown. (**C**) Serial PMCAb reactions were seeded with 10% brain or spleen homogenates from hamsters inoculated with dsPMCAb- or PMCAb-derived material, subjected to four serial rounds and reaction products were analyzed by Western blot. As positive controls, serial PMCAb reactions were seeded with 10^9^-fold diluted SSLOW brain material. As a negative control for cross-contamination, non-seeded serial PMCAb reactions were conducted in parallel (NS). Lane “PMCAb” refers to PMCAb reactions seeded with brain and spleen tissue from animal #8 in panel **A** “PMCAb-derived”. Independent serial PMCAb reactions were repeated twice for each samples and produced identical results. Black and white triangles mark diglycosylated and monoglycosylated glycoforms, respectively, whereas arrows mark the unglycosylated form. All blots were stained with 3F4 antibody.

Syrian hamsters were inoculated IC with brain-, PMCAb-, dsPMCAb- or rsPMCA-derived SSLOW. The amounts of PK-resistant material in the 10-fold diluted PMCAb-, dsPMCAb- or rsPMCAb-derived samples used for inoculation was higher than the amounts of PrP^Sc^ in 10^4^-fold diluted but lower than that in 10^3^-fold diluted SSLOW brain material (Supplementary Figure 1). Both 10^3^-fold and 10^4^-fold diluted SSLOW brain material was used as references. All animals inoculated with brain-derived SSLOW developed clinical signs of disease typical for SSLOW (listed in Materials and Methods) and progressed to the terminal stage by 503–609 days post inoculation, at which time they were euthanized (Table 1). All animals showed slow progression of clinical disease typical for the SSLOW strain. None of the animals inoculated with PMCAb-, dsPMCAb- or rsPMCA-derived SSLOW showed obvious clinical signs and were euthanized at 614 or 621 days post inoculation (Table 1). Nevertheless, analysis of brain and spleen materials revealed that animals inoculated with PMCAb- or rsPMCAb-derived SSLOW were PrP^Sc^-positive as judged by the presence of a PK-resistant signal on Western blot. In the group inoculated with PMCAb-derived SSLOW, seven out of eight animals showed PrP^Sc^ in their brain and all eight animals had PrP^Sc^ in their spleen (Fig. 2A, Table 1). Brains and spleens from all five animals inoculated with rsPMCAb-derived SSLOW were positive for PrP^Sc^ (Fig. 2A, Table 1). In contrast, none of the six animals inoculated with dsPMCA-derived SSLOW showed PrP^Sc^ signal in their brains or spleens.

Lack of a signal in the group inoculated with dsPMCAb-derived SSLOW could be due to changes in strain properties and accumulation of predominantly PK-sensitive PrP^Sc^. To test this possibility, brain materials from PMCAb, dsPMCAb and rsPMCA animal groups, as well as normal animals 600–670 days of age, were treated with increasing concentrations of PK starting at 2 μg/ml. As expected, animals from PMCAb and rsPMCA groups showed a consistent signal in the whole range of PK concentrations tested, from 2 μg/ml to 100 μg/ml (Fig. 2B). In contrast, no signal was detected in brains from the dsPMCAb group or brains of aged animals even at the lowest concentration of PK (Fig. 2B). In fact, the PK-resistance profile was indistinguishable between dsPMCAb-inoculated animals and the normal aged group.

To test whether miniscule amounts of PrP^Sc^ undetectable by Western blot were present in the animals inoculated with dsPMCA-derived SSLOW, brain and spleen materials from this group were subjected to serial PMCAb. Previously, serial PMCAb or PMCA was shown to be capable of detecting single PrP^Sc^ particles in several strains, including SSLOW^4^,^26^,^27^. In parallel, brain and spleen material from animal #8 in the group inoculated with PMCAb-derived SSLOW was also subjected to serial PMCAb. Serial PMCAb reactions seeded with 10^9^-fold diluted SSLOW brain material were used as a positive control, whereas non-seeded reactions were conducted as negative controls. Four serial rounds of PMCAb were performed according to the protocol that we previously determined to be sufficient for detecting limiting dilutions of SSLOW^4^. As expected, 10^9^-fold diluted SSLOW brain material, as well as the brain and spleen materials from the animal #8 inoculated with PMCAb-derived SSLOW, all showed positive signals after serial PMCAb (Fig. 2C). These results confirmed that animal #8 was infected and that limiting dilutions of SSLOW could be detected. In contrast, brains and spleens from all six animals inoculated with dsPMCA-derived SSLOW were negative on Western blot after serial PMCAb (Fig. 2C). This result established that in the animals inoculated with dsPMCA-derived SSLOW, any detectible prion infection was cleared. Taken together, these data suggest the ability to infect a host strongly depends on the sialylation status of PrP^Sc^.

**Figure 3:**
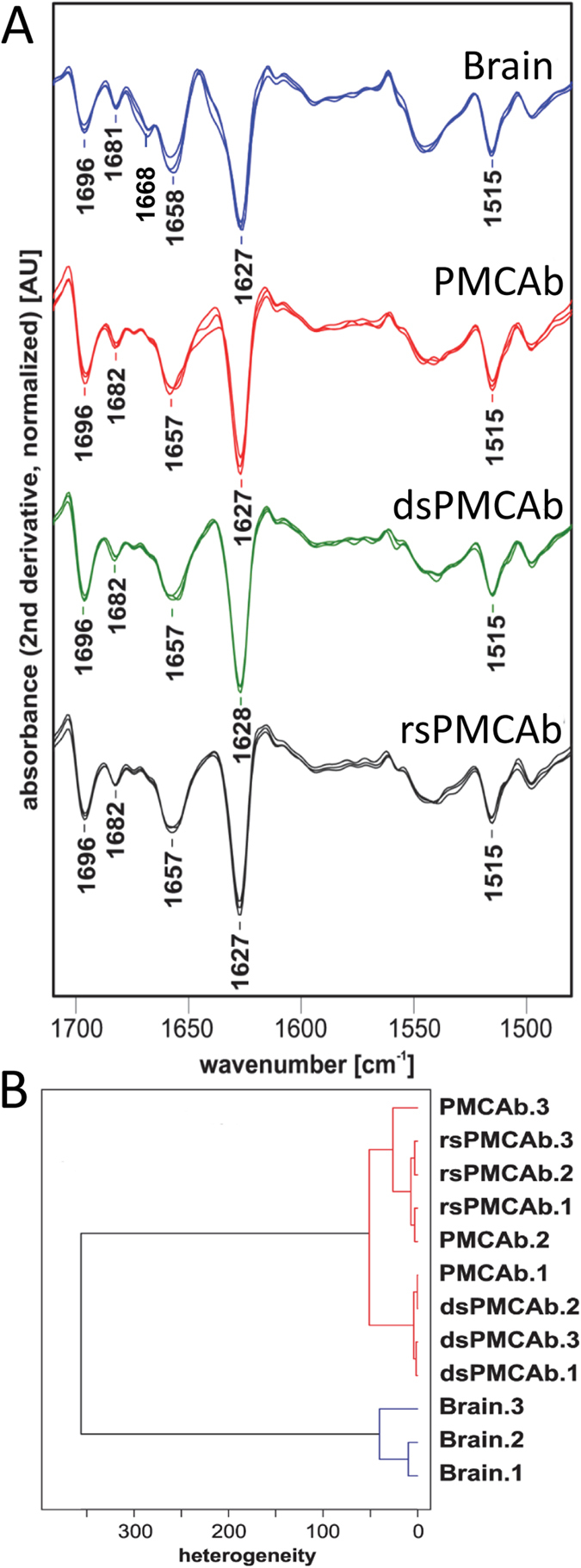
Assessing secondary structure by infrared microspectroscopy. (**A**) IR spectra obtained from PrP^Sc^ material purified from brains of three Syrian Hamster infected with SSLOW (blue), or PMCAb- (red), dsPMCAb- (green) or rsPMCAb-derived SSLOW (black). IR spectra were collected for products of three independent reactions for each format, PMCAb, dsPMCAb or rsPMCAb. Each spectrum represents a min/max normalized (tyrosine band at 1515 cm^−1^) second derivative spectrum obtained by averaging ten individual point spectra. (**B**) Dendrogram analysis of conformational heterogeneity of SSLOW PrP^Sc^ material purified from brains of animals infected with SSLOW or PMCAb, dsPMCAb, or rsPMCAb reactions (n = 3 independent brains or reactions). Dendrogram obtained by hierarchical cluster analysis of the mean microspectra using the information content in the amide I region (1620–1690 cm^−1^), using the *D* value as the interspectral distance measure, and Ward’s algorithm as the clustering method.

Theoretically, reversible changes of the PrP^Sc^ secondary or tertiary structure during dsPMCAb and rsPMCAb could account for the loss and restoration of infectivity, respectively. In fact, previous studies that employed infrared microscopy revealed conformational differences between 263K scrapie brain material and 263K-seeded PMCA-derived products^28^. To test whether this is the case, PrP^Sc^ was purified from SSLOW brain-, PMCAb-, dsPMCAb- and rsPMCAb-derived materials and analyzed by infrared microspectroscopy (IR-MSP). Brain-derived SSLOW showed a peak at 1627 cm^−1^ that corresponds to a β-sheet rich conformation, a small peak at 1696 cm^−1^ that also reports on β-sheet structures and a peak at 1658 cm^−1^ which is conventionally assigned to an α-helical conformation (Fig. 3A). The association between specific IR absorption bands at 1658 cm^−1^ and α-helical structure elements in PrP^Sc^ is currently under discussion^29^. Analysis of PMCAb-derived materials confirmed that subtle structural changes occurred in PrP^Sc^ during serial amplification. Specifically, the minor peak visible in brain-derived SSLOW at 1668 cm^−1^ disappeared in PMCAb-, dsPMCAb- and rsPMCAb SSLOW. (Figure 3A). Remarkably, the spectra of PMCAb-, dsPMCAb- and rsPMCAb-derived SSLOW were undistinguishable between each other illustrating that PrP^Sc^ structure is not altered by the changes in sialylation status. Nevertheless, to determine whether very minor structural differences between PMCAb, dsPMCAb and rsPMCAb groups exist, hierarchical cluster analysis was performed (Fig. 3B). Previously, the hierarchical cluster analysis was found to be very helpful in detecting similarities or dissimilarities in the conformation sensitive amide I region of IR spectra of prion strains, isolates or their PMCA derivatives that were not obvious by visual inspection of spectra^28^,^30^. The analysis revealed two main clusters: spectra of three SSLOW brains belong to one cluster, whereas all spectra of PMCAb-, dsPMCAb- and rsPMCAb-derived materials formed another cluster. While the structural differences between the two clusters were clearly noticeable, the structural heterogeneity between PMCAb-, dsPMCAb- and rsPMCAb-derive products were of the same degree as heterogeneity between three SSLOW brains. In summary, IR-MSP and cluster analysis demonstrated that minor conformational changes in PrP^Sc^ structure did, indeed, occur during PMCAb and might account for some loss of infectivity in PMCAb-derived SSLOW when compared to the brain-derived SSLOW. However, the lack of notable structural differences between PMCAb-, dsPMCAb- and rsPMCAb-derived SSLOW PrP^Sc^ argues that the conformation of amplification products was not affected by sialylation status.

## Discussion

The current study supports the hypothesis that the ability of prions to infect a host is controlled by the sialylation status of PrP^Sc^ 14,15. According to this hypothesis removal of terminal sialic acid residues exposes galactose that serves as an “eat me” signal for macrophages and microglia^15^. Both PMCAb and rsPMCAb-derived materials infected the host as judged by presence of PrP^Sc^ in brain and spleen, whereas animals inoculated with dsPMCAb-derived material showed no signs of prion infection. The possibility exists that dsPMAb material produced a new prion strain characterized by deposition of predominantly PK-sensitive PrP^Sc^ and lacked clinical symptoms. However, this hypothesis was not supported by the experiment that employed low concentrations of PK. In fact, the PK resistance profiles of brain material from the dsPMCAb group and aged animals were identical. Moreover, considering that PMCAb, dsPMAb and rsPMCAb material all showed indistinguishable PrP^Sc^ conformations, the notion that dsPMCAb material results in a new strain different from that of PMCAb and rsPMCAb would challenge the current paradigm regarding the relationship between PrP^Sc^ structure and strain identity. Because the SSLOW materials produced in PMCAb, dsPMAb or rsPMCAb all showed indistinguishable conformations, regardless of their sialylation status, the loss of infectivity of dsPMCAb-derived SSLOW can be attributed to its reduced sialylation status. Remarkably, the loss of infectivity could be restored to the level observed for PMCAb-derived SSLOW, when the sialylation of dsPMCAb-derived SSLOW was restored in rsPMCAb reactions to the level typical for PMCAb-derived products. This result established a cause-and-effect relationship between sialylation status of PrP^Sc^ and its infectivity. These findings open new opportunities for therapeutic intervention via manipulating metabolic pathways that regulate the sialylation status of PrP^Sc^.

Previous studies produced highly infectious PrP^Sc^ *in vitro* using recombinant PrP^31^,^32^,^33^. Because entire N-linked glycans were missing in PrP^Sc^ produced from recombinant PrP, the “eat me” signal in the form of exposed galactose was also missing. For this reason, it is unlikely that the innate immune system and/or microglia could identify PrP^Sc^ of synthetic origin as potential pathogens in the same manner as it might deal with asialo-PrP^Sc^ with displays an “eat me” signal. Notably, lack of PrP^Sc^ in spleens from the dsPMCAb group observed in the current study suggests that PrP^Sc^ with reduced sialylation status did not spread to SLOs or that it spread there but was effectively neutralized by the immune system.

The infectivity of PMCAb-derived SSLOW was lower in comparison to the brain-derived SSLOW. The decline in prion infectivity during serial PMCAb or PMCA reactions was observed in previous studies^14^,^34^. As documented by IR-MSP, this loss could be attributed to the changes in PrP^Sc^ conformation, even though these were found to be very subtle (Fig. 3A). Nevertheless, even modest differences were sufficient to prolong the incubation time to disease beyond the animal’s normal life span (Table 1). Similar trends in structure and infectivity were previously observed for 263 K subjected to serial PMCA or PMCAb^14^,^28^. Shifting the conformation of PrP^Sc^ via PMCAb might offer a valuable approach for defining elements of PrP^Sc^ structure essential for prion infectivity in future studies.

The long incubation time associated with SSLOW strain, together with a decline in infectivity attributed to PMCAb procedure, poses a couple of questions regarding the extent to which sialylation controls prion infectivity. Would desialylation work to the same extent for a strain with a short incubation time? Would desialylation completely destroy the infectivity of scrapie material with a high infectivity titer? Our previous studies conducted with 263K, for which the incubation time ranges from 60 to ~170 days depending on the dose^35^, displayed similar effects. No clinical diseases or PrP^Sc^ were found in animals inoculated IC with dsPMCAb-derived 263K for at least 342 days post infection, when animals were euthanized^14^. The second question would be difficult to address until a novel approach for generating desialylated PrP^Sc^ is developed. We found that treatment of non-denatured PrP^Sc^ with commercially available sialidases was not effective with respect to desialylation presumably due to the aggregated nature of the scrapie material.

Sialylation of glycoproteins in a cell is controlled by two groups of enzymes sialyltransferases and sialidases^36^,^37^. Because PrP^Sc^ arises from PrP^C^ via changes in its conformation, manipulating the sialylation status of PrP^C^ offers the primary mechanism by which sialylation of PrP^Sc^ could be controlled. Our previous work demonstrated that knocking out sialidases that are expressed in CNS (*Neu1, Neu3* or *Neu4*) or application of general sialidase inhibitors did not change the steady-state sialylation levels of PrP^C^ in brain or cultured cells, respectively^38^. These data suggest that desialylated PrP^C^ molecules are degraded very fast and do not contribute to the steady-state pool of PrP^C^. In contrast, a general inhibitor of sialyltransferases was found to reduce the sialylation levels of PrP^C^ in N2a cells, suggesting that manipulating the activity of sialyltransferases offers a more effective strategy than that of sialidases for controlling sialylation status of PrP^C^ 38. The second mechanism that defines the sialylation status of PrP^Sc^ is strain-specific and involves selective recruitment of sialoglycoforms of PrP^C^ by PrP^Sc^ 24. Our recent studies revealed that heavily sialylated PrP^C^ was preferentially excluded from conversion, and the degree of exclusion was strain-specific^24^,^39^. The structure of PrP^Sc^ appears to define the maximum density of sialic acid residues that can be accommodated within given strain-specific structures. The third mechanism is related to the tissue-specific differences in sialylation levels of PrP^Sc^ 25. Regardless of prion strain, PrP^Sc^ deposited in secondary lymphoid organs was found to be more sialylated than brain-derived PrP^Sc^ 25. Enhanced sialylation of spleen-derived PrP^Sc^ was attributed to post-conversion sialylation, i.e. direct sialylation of PrP^Sc^ by sialyltransferases^25^.

Sialylation along with other molecular cues is used by the mammalian innate immune system for discriminating “self” from “non-self” or “altered self”^5^,^6^. The surfaces of all mammalian cell types are covered with sialic acid residues^40^, which act as a part of “self-associated molecular pattern”. In contrast, the majority of microbial pathogens lack sialic acid residues on their glycans, which instead leave galactose exposed^5^,^6^. Terminal galactoses serve as part of a “pathogen-associated molecular pattern” generating “eat me” signals for professional and non-professional macrophages^5^. Moreover, a decline in the density of sialic residues on cell surfaces represents one of the molecular signatures of “apoptotic-cell-associated molecular patterns” observed in apoptotic or aging cells^41^,^42^. Erythrocytes and platelets that lose sialic acid residues due to aging are phagocytosed by Kupffer cells^43^,^44^. The functions fulfilled by the cells of innate immune system in the periphery are performed in CNS by microglia, astrocytes, endothelial cells and other cell types^16^. Recent studies showed that neurites with reduced surface sialylation are opsonized by the complement component C1q and cleared by microglia, whereas sialylation of neurites inhibited complement-mediated clearance^7^,^8^.

To fulfill its function, the innate immune system involves diverse mechanisms and relies on several classes of molecules, which recognize sialo- or asialoglycanson cell surfaces. Asialoglycoprotein receptors are expressed by Kupffer cells and microglia and bind glycans with exposed terminal galactoses, helping to clear aged cells or asialoglycoprotein complexes^45^. Galectins, a family of secreted protein receptors, are expressed in the periphery and CNS and recognize complex patterns composed of sialo- or asialo-glycans^46^. An alternative mechanism that relies on glycan recognition involves the lectin pathway of the complement system that include factors C1q and C3^47^. Both C1q and C3 are expressed in a brain by glial and neuronal cells^48^,^49^. While the complement system is supposed to defend against pathogens, C1q and C3 were found to assist in the trafficking and accumulation of prions in secondary lymphoid organs at early stages of infection upon administration of prions via peripheral routes^50^,^51^. Genetic or pharmacological depletions of C3 or its receptor CD21/35 were found to prolong incubation time to disease^50^,^51^,^52^,^53^. Nevertheless, the role of C1q and C3 in sporadic prion disease or diseases transmitted via the IC route is not clear. Upon administration of prions IC, C1q and C3 were found to be expressed at high levels in brain during disease progression and were shown to be associated with neurons, astrocytes and microglia^48^. However, factor H balances overstimulation of the complement system by binding to terminal sialic acids and conferring protection to healthy mammalian cells by preventing over activation of C1q^47^,^54^. It would be important to determine if any of these known mechanisms are involved in the lack of infectivity of PrP^Sc^ with low sialylation levels in future studies.

## Conclusions

The current work established that the ability of prions to infect a host via IC administration crucially depends on the PrP^Sc^ sialylation status. Remarkably, PrP^Sc^ infectivity could be switched off and on in a reversible manner by removing and reinstalling PrP^Sc^ sialylation, respectively. In the periphery, sialylation acts as a part of a “self-associated molecular pattern” helping the immune system to recognize cells of mammalian organism and set them apart from pathogens. The current work suggests that cells that perform the functions of the innate immune system in the CNS utilize a similar sialylation-based strategy for discriminating between “self” and “non-self”.

## Methods

### Ethics statement

This study was carried out in strict accordance with the recommendations in the Guide for the Care and Use of Laboratory Animals of the National Institutes of Health. The animal protocol was approved by the Institutional Animal Care and Use Committee of the University of Maryland, Baltimore (Assurance Number A32000-01; Permit Number: 0215002).

### Protein misfolding cyclic amplification with beads (PMCAb)

10% normal brain homogenate (NBH) from healthy hamsters was prepared as described previously^55^. To produce desialylated substrates, 10% NBH was treated with *Arthrobacter ureafaciens* (cat #P0722L, New England Biolabs, Ipswich, MA) sialidase that has broad substrate specificity, as follows. After preclearance of NBH at 500 × *g* for 2 min, the enzyme buffer supplied by the manufacturer was added to the supernatant using manufacturer’s instructions, followed by 200 units/mL of sialidase, and incubated on a rotator at 37 °C for 5 h. The resulting material is referred to as dsNBH substrate used in dsPMCAb, a PMCAb format conducted using dsNBH.

PMCAb and dsPMCAb reactions were conducted as previously described^14^,^22^, using a Misonix S-4000 microplate horn (Qsonica LLC, Newtown, CT) in the presence of two 2/32″ Teflon beads in each tube (McMaster-Carr, Elmhurst, IL). Both types of reactions were seeded with 10^3^-fold diluted SSLOW scrapie brain homogenates. To produce PMCAb-derived material, 24 serial rounds with 10-fold dilution between rounds were conducted using NBH as a substrate. To produce dsPMCAb material, 7 serial rounds with 1,000-fold dilution between rounds were conducted with dsNBH substrate. To produce resialylated SSLOW PrP^Sc^, 10 serial rounds with 10-fold dilution between rounds were conducted using NBH as a substrate and dsPMCAb-derived SSLOW as seeds (this reaction format will be referred to as resialylated or rsPMCAb). Prior to inoculation, PMCAb-, dsPMCAb- and rsPMCAb-derived products were diluted an additional 10-fold.

To test for the presence of PrP^Sc^ in the brains or spleens of hamsters inoculated with PMCAb- and dsPMCAb-derived materials, 90 μl of substrate was supplied with two 2/32″ Teflon beads and seeded with 10 μl of 10% brain or spleen homogenates. Four serial rounds were conducted with 10-fold dilution of the reaction products into fresh substrate between rounds. 10^9^-fold diluted 10% brain homogenate from a hamster terminally ill with SSLOW was used as a positive control and non-seeded serial PMCAb reactions as negative controls. In all PMCAb, dsPMCAb or rsPMCAb reactions, one round consisted of 20 sec sonication pulses delivered at 170 W energy output applied every 20 min during a 24 hour period.

### Animal bioassay

Weanling Golden Syrian hamsters were inoculated IC with 50 μl 10^3^- and 10^4^-fold diluted SSLOW scrapie brain homogenates, 10-fold diluted PMCAb, dsPMCAb or rsPMCAb-derived materials under 2% O_2_/4 minimum alveolar concentration (MAC) isoflurane anesthesia. After inoculation, animals were observed daily for disease using a ‘blind’ scoring protocol and euthanized at the terminal stage of disease. The following symptoms typical for SSLOW were observed in animals inoculated with SSLOW brain material: a non-habituating startle response to sound and touch; an agitated, fidgeting behavior; pear shaped body; increasingly squat, paddling gait; and dry, patchy and shedding hair. After the first symptoms were observed, it took 80–120 days for the diseases to progress to a terminal stage. Animals that did not develop clinical signs of disease were euthanized at 614 or 621 days post inoculation (Table 1).

### 2D Western blotting

Samples of 25 μL prepared in loading buffer as described in “Western blot” section below were solubilized for 1 h at room temperature in 200 μL solubilization buffer (8 M Urea, 2% (wt/vol) CHAPS, 5 mM TBP, 20 mM Tris pH 8.0), alkylated by adding 7 μL of 0.5 M iodoacetamide and incubated for 1 h at room temperature. Then, 1150 μL of ice-cold methanol was added, and samples were incubated for 2 h at −20 °C. After centrifugation at 16,000 g at 4 °C, supernatant was discarded, and the pellet was re-solubilized in 160 μL rehydration buffer (7 M urea, 2 M thiourea, 1% (wt/vol) DTT, 1% (wt/vol) CHAPS, 1% (wt/vol) Triton X-100, 0.4% (vol/vol) ampholyte, trace amount of Bromophenol Blue). Fixed pre-cast IPG (immobilized pH gradient) strips (cat. #ZM0018, Life Technologies, Carlsbad, CA) with a linear pH gradient 3–10 were rehydrated in 155 μL of the resulting mixture overnight at room temperature inside IPG Runner cassettes (cat. #ZM0003, Life Technologies). Isoelectrofocusing (first dimension separation) was performed at room temperature with rising voltage (175 V for 15 minutes, then 175–2,000 V linear gradient for 45 minutes, then 2,000 V for 30 minutes) with a Life Technologies Zoom Dual Power Supply, using the XCell SureLock Mini-Cell Electrophoresis System (cat. #EI0001, Life Technologies). The IPG strips were then equilibrated for 15 minutes consecutively in (i) 6 M Urea, 20% (vol/vol) glycerol, 2% SDS, 375 mM Tris-HCl pH 8.8, 130 mM DTT and (ii) 6 M Urea, 20% (vol/vol) glycerol, 2% SDS, 37 5 mM Tris-HCl pH 8.8, 135 mM iodoacetamide and loaded on 4–12% Bis-Tris ZOOM SDS-PAGE pre-cast gels (cat. #NP0330BOX, Life Technologies). For the second dimension, SDS-PAGE in MES-SDS buffer (cat. # B0002, Life Technologies, Carlsbad, CA) was performed for 1 h at 170 V. Immunoblotting was performed as described elsewhere and stained using 3F4 antibody (cat. #SIG-39600, Covance).

2D Western blot signal intensity was digitized for densitometry analysis using AlphaView software (ProteinSimple, San Jose, CA). For generating sialylation profiles for diglycosylated isoforms, densitometry analysis of 2D blots was performed using the “Lane profile” function in the AlphaView program, the highest curve signal value was taken as 100%.

### Procedure for purification of scrapie material

Extraction of PrP^Sc^ from brain tissue, PMCAb, dsPMCAb and rsPMCAb reactions for FTIR microspectroscopic analysis were performed as described by Daus *et al*.^28^ with the following modifications for brain tissue: hemispheres of mid-sagitally split hamster brains (approximately 0.5 g) were each homogenizedin 10 ml of homogenization buffer. Brain tissue homogenates were subjected to the extraction procedure, and five final pellets of highly purified PrP^Sc^ were obtained from one hemisphere. For infrared spectroscopic analysis final PrP^Sc^ pellets were washed as described^28^ and resuspended in 10 μl of double-distilled H_2_O. 1 μl aliquots of these PrP^Sc^ suspensions were transferred for drying onto a CaF_2_ window of 1 mm thickness (Korth Kristalle GmbH, Altenförde, Germany).

### Infrared microspectroscopy

For brain-, PMCAb-, dsPMCAb- and rsPMCAb-derived scrapie material, three independent preparations each were obtained and characterized by infrared microspectroscopy (IR-MSP). IR-MSP measurements were carried out as previously described^28^. Briefly, mid-IR spectra were acquired in transmission mode using an IFS 28/B FT-IR spectrometer from Bruker (Bruker Optics GmbH, Ettlingen Germany) that was linked to an IRscope II infrared microscope (Bruker). IR microspectra were recorded with a spatial resolution of approximately 80 μm. Nominal spectral resolution was 4 cm^−1^, and the zero filling factor was 4. For each background and for each sample spectrum, 512 individual interferograms were averaged and apodized using a Blackman-Harris 3-term apodization function. To attain an improved signal-to-noise ratio (SNR) and to address the aspect of within-sample heterogeneity, 10 point spectra were acquired and averaged per purified PrP^Sc^ sample. Data acquisition and spectral preprocessing was carried out by utilizing Bruker’s instrument software OPUS v. 5.5. Second derivative spectra were obtained by means of a 9-smoothing point Savitzki-Golay derivative filter. All derivative spectra were min/max normalized to the tyrosine band at 1515 cm^−1^.

### Cluster analysis

Unsupervised hierarchical cluster analysis was used to reveal groupings in the IR microspectra. The analysis was performed using the cluster analysis function of the OPUS data acquisition software with mean spectra from three independent preparations for each group as inputs. Pre-processing of the mean spectra involved application of a Savitzky-Golay second derivative filter with 13 smoothing points^56^. Inter-spectral distances were calculated using the information from the secondary structure-sensitive amide I region (1620 and 1690 cm^−1^) as so-called D-values - scaled Pearson’s correlation coefficients^57^ - while Ward’s algorithm^58^ was used for hierarchical clustering.

### Proteinase K digestion and Western blot

10 μL of 10% spleen homogenates or 1% brain homogenates were mixed with an equal volume of 4% sarcosyl in PBS, supplemented with 50 mM Tris, pH 7.5, and digested with 25 μg/ml PK (cat. #P8107S, New England Biolabs, Ipswich, MA) for 30 min at 37 °C. 10 μL aliquots of PMCAb-, dsPMCAb- or rsPMCAb-derived materials were supplemented with 5 μL 1% SDS and 5 μL PK to final concentrations of 0.25% SDS and 25 μg/mL PK, and incubated at 37 °C for 1 hour. Then, SDS sample buffer was added, samples were boiled for 10 minutes and loaded onto NuPage 12% BisTris gel (cat. #NP0341BOX, Life Technologies, Carlsbad, CA), then transferred to PVDF membrane, and probed with 3F4 (cat. #SIG-39600, Covance).

To analyze sensitivity to PK, 10% brain homogenates were diluted 10-fold with 2% sarcosyl in PBS and supplied with increasing concentrations of PK to final concentrations of 0, 2, 10, 50 or 100 μg/ml. After incubation with shaking at 37 °C for 30 min, the reactions were stopped by addition of 4x SDS-sample buffer and boiling the samples for 10 min. Samples were loaded onto NuPAGE 12% BisTris gels, transferred to PVDF membrane, and detected with 3F4 antibody.

## Additional Information

**How to cite this article**: Katorcha, E. *et al*. Reversible off and on switching of prion infectivity via removing and reinstalling prion sialylation. *Sci. Rep.* **6**, 33119; doi: 10.1038/srep33119 (2016).

## Supplementary Material

Supplementary Information

## Figures and Tables

**Table 1:** Bioassay of SSLOW samples in golden Syrian hamsters.

SSLOW inocula	n_s_/n_t_*	n_PrPSc_/n_t_** brain	n_PrPSc_/n_t_** spleen	Euthanized at days post-inoculation
10^3^ fold diluted brain-derived	7/7	7/7	N/A	503, 503, 503, 503, 503, 591, 609
10^4^ fold diluted brain-derived	7/7	7/7	N/A	504, 540, 551, 552, 556, 560, 577
PMCAb-derived	0/8	7/8	8/8	621
dsPMCAb-derived	0/6	0/6	0/6	614
rsPMCAb-derived	0/5	5/5	5/5	614

^*^Number of animals with clinical signs (n_s_) over the total number of animals (n_t_) that survived to the end of the experiment.

^**^Number of animals with PrP^Sc^ in brain homogenates or spleen homogenates (n_PrPSc_) over the total number of animals survived to the end of the experiment (n_t_).

## References

[b1] Prusiner S. B. Novel proteinaceous infectious particles cause scrapie. Science 216, 136–144 (1982). 6801762 10.1126/science.6801762

[b2] Miller M. W. & Williams E. S. Chronic wasting disease of cervids. Curr. Top. Microbiol. Immunol. 284, 193–214 (2004). 15148993 10.1007/978-3-662-08441-0_8

[b3] Brown P. & Gajdusek D. C. The human spongiform encephalopathies: kuru, Creutzfeldt-Jakob disease, and the Gerstmann-Sträussler-Scheinker syndrome. Curr. Top. Microbiol. Immunol. 172, 1–20 (1991). 1687378 10.1007/978-3-642-76540-7_1

[b4] Makarava N., Savtchenko R., Alexeeva I., Rohwer R. G. & Baskakov I. V. Fast and ultrasensitive method for quantitating prion infectivity titer. Nature Commun. 3, 741 (2012). 22415832 10.1038/ncomms1730 PMC3518416

[b5] Varki A. Sialic acids in human health and disease. Trends in Molecular Medicine 14, 351–360 (2008). 18606570 10.1016/j.molmed.2008.06.002 PMC2553044

[b6] Varki A. Since there are PAMPs and DAMPs, there must be SAMPs? Glycan “self-associated molecular patterns” dampen innate immunity, but pathogens can mimic them. Glycobiology 21, 1121–1124 (2011). 21932452 10.1093/glycob/cwr087 PMC3150115

[b7] Linnartz B., Kopatz J., Tenner A. J. & Neumann H. Sialic acid on the neuronal glycocalyx prevents complement C1 binding and complement receptor-3-mediated removal by microglia. J. Neurosci 32, 946–952 (2012). 22262892 10.1523/JNEUROSCI.3830-11.2012 PMC4037907

[b8] Linnartz-Gerlach B., Schuy C., Shahraz A., Tenner A. J. & Neumann H. Sialylation of neurites inhibits complement-mediated macrophage removal in a human macrophage-neuron Co-Culture System. Glia 64, 35–47 (2016). 26257016 10.1002/glia.22901 PMC4715670

[b9] Turk E., Teplow D. B., Hood L. E. & Prusiner S. B. Purification and properties of the cellular and scrapie hamster prion proteins. Eur J Biochem 176, 21–30 (1988). 3138115 10.1111/j.1432-1033.1988.tb14246.x

[b10] Endo T., Groth D., Prusiner S. B. & Kobata A. Diversity of oligosaccharide structures linked to asparagines of the scrapie prion protein. Biochemistry 28, 8380–8388 (1989). 2574992 10.1021/bi00447a017

[b11] Stimson E., Hope J., Chong A. & Burlingame A. L. Site-specific characterization of the N-linked glycans of murine prion protein by high-performance liquid chromatography/electrospray mass spectrometry and exoglycosidase digestions. Biochemistry 38, 4885–4895 (1999). 10200178 10.1021/bi982330q

[b12] Stahl N. . Structural studies of the scrapie prion protein using mass spectrometry and amino acid sequencing. Biochemistry 32, 1991–2002 (1993). 8448158 10.1021/bi00059a016

[b13] Rudd P. M. . Glycosylation differences between the normal and pathogenic prion protein isoforms. Proc Natl Acad Sci USA 96, 13044–13049 (1999). 10557270 10.1073/pnas.96.23.13044 PMC23897

[b14] Katorcha E., Makarava N., Savtchenko R., D’Azzo A. & Baskakov I. V. Sialylation of prion protein controls the rate of prion amplification, the cross-species barrier, the ratio of PrPSc glycoform and prion infectivity. PLOS Pathog. 10, e1004366, 10.1371/journal.ppat.1004366 (2014). 25211026 PMC4161476

[b15] Baskakov I. V. & Katorcha E. Multifaceted role of sialylation in prion diseases. Front. Neurosci. 10, 358, 10.3389 (2016). 27551257 10.3389/fnins.2016.00358 PMC4976111

[b16] Lampron A., Elali A. & Rivest S. Innate Immunity in the CNS: Redefining the Relatiionship between the CNS and Its Environment. Neuron 78, 214–232 (2013). 23622060 10.1016/j.neuron.2013.04.005

[b17] Abt M. C. & Pamer E. G. Commensal bacteria mediated defences against pathogens. Current Opinion in Immunology 29, 16–22 (2014). 24727150 10.1016/j.coi.2014.03.003 PMC4132187

[b18] Abt M. C. . Commensal Bacteria Calibrate the Activation Threshold of Innate Antiviral Imuunity. Immunity 37, 158–170 (2012). 22705104 10.1016/j.immuni.2012.04.011 PMC3679670

[b19] Makarava N. . Recombinant prion protein induces a new transmissible prion disease in wild type animals. Acta Neuropathol. 119, 177–187 (2010). 20052481 10.1007/s00401-009-0633-x PMC2808531

[b20] Makarava N. . Stabilization of a prion strain of synthetic origin requires multiple serial passages. J Biol Chem 287, 30205–30214 (2012). 22807452 10.1074/jbc.M112.392985 PMC3436274

[b21] Jeffrey M. . Pathology of SSLOW, a transmissible and fatal synthetic prion protein disorder, and comparison with naturally occurring classical transmissible spongoform encephalopathies. Neuropath.Appl.Neurobiol. 40, 296–310 (2014). 10.1111/nan.12053 PMC3783560 23578208

[b22] Gonzalez-Montalban N. . Highly Efficient Protein Misfolding Cyclic Amplification. PLoS Pathogen 7, e1001277 (2011). 21347353 10.1371/journal.ppat.1001277 PMC3037363

[b23] Gonzalez-Montalban N., Makarava N., Savtchenko R. & Baskakov I. V. Relationship between Conformational Stability and Amplification Efficiency of Prions. Biochemistry 50, 7933–7940 (2011). 21848309 10.1021/bi200950v PMC3183828

[b24] Katorcha E., Makarava N., Savtchenko R. & Baskakov I. V. Sialylation of the prion protein glycans controls prion replication rate and glycoform ratio. Sci Rep 5, 16912, 10.1038/srep16912 (2015). 26576925 PMC4649626

[b25] Srivastava S. . Post-conversion sialylation of prions in lymphoid tissues. Proc Acad Natl Sci USA 112, E6654–6662, 10.1073/pnas.1517993112 (2015). PMC4672809 26627256

[b26] Saa P., Castilla J. & Soto C. Ultra-efficient Replication of Infectious Prions by Automated Protein Misfolding Cyclic Amplification. J.Biol.Chem. 281, 35245–35252 (2006). 16982620 10.1074/jbc.M603964200

[b27] Pritzkow S. . Quantitative detection and biological propagation of scrapie seeding activity *in vitro* facilitate use of prions as model pathogens for disinfection. Plos ONE 6, e20384 (2011). 21647368 10.1371/journal.pone.0020384 PMC3103549

[b28] Daus M. L. . Infrared Microspectroscopy Detects Protein Misfolding Cyclic Amplification (PMCA)-Induced Conformational Alterations in Hamster Scrapie Progeny Seeds. J Biol Chem 288, 35068–35080 (2013). 24163371 10.1074/jbc.M113.497131 PMC3853259

[b29] Smirnovas V. . Structural organization of brain-derived mammalian prions examined by hydrogen-deuterium exchange. Nat Struct Mol Biol 18, 504–506 (2011). 21441913 10.1038/nsmb.2035 PMC3379881

[b30] Spassov S., Beekes M. & Naumann D. Structural differences between TSEs strains investigated by FT-IR spectroscopy. Biochim Biophys Acta 1760, 1138–1149 (2006). 16730908 10.1016/j.bbagen.2006.02.018

[b31] Wang F., Wang X., Yuan C. G. & Ma J. Generating a Prion Bacterially Expressed Recombinant Prion Protein. Science 327, 1132–1135 (2010). 10.1126/science.1183748 PMC2893558 20110469

[b32] Deleault N. R. . Isolation of phosphatidylethanolamine as a solitary cofactor for prion formation in the absence of nucleic acids. Proc Acad Natl Sci USA 109, 8546–8551 (2012). 10.1073/pnas.1204498109 PMC3365173 22586108

[b33] Deleault N. R. . Cofactor molecules maintain infectious conformation and restrict strain properties in purified prions. Proc.Acad.Natl.Sci.USA 109, E1938–E1946 (2012). 10.1073/pnas.1206999109 PMC3396481 22711839

[b34] Klingeborn M., Race B., Meade-White K. D. & Chesebro B. Lower specific infectivity of protease-resistant prion protein generated in cell-free reactions. Proc Acad Natl Sci USA 108, E1244–1253 (2011). 10.1073/pnas.1111255108 PMC3228482 22065744

[b35] Kimberlin R. H. & Walker C. A. Pathogenesis of scrapie (strain 263K) in hamsters infected intracerebrally, intraperitoneally or intraocularly. J.Gen.Virol. 67, 255–263 (1986). 3080549 10.1099/0022-1317-67-2-255

[b36] Miyagi T. & Yamaguchi K. Mammalian sialidases: physiological and pathological roles in cellular functions. Glycobiology 22, 880–896 (2012). 22377912 10.1093/glycob/cws057

[b37] Audry M. . Current trend in the structure-activity relationships of sialylatransferases. Glycobiology 21, 716–726 (2011). 21098518 10.1093/glycob/cwq189

[b38] Katorcha E. . Knocking out of cellular neuraminidases Neu1, Neu3 or Neu4 does not affect sialylation status of the prion protein. PLoS One 10, e0143218, 10.1371/journal.pone.0143218 (2015). 26569607 PMC4646690

[b39] Makarava N., Savtchenko R. & I.V. B. Two alternative pathways for generating transmissible prion disease de novo. Acta Neuropathologica Communications 3, 69, 10.1186/s40478-015-0248-5 (2015). 26556038 PMC4641408

[b40] Collins B. E. . Masking of CD22 by cis ligands does not prevent redistribution of CD22 to sites of cell contact. Proc Acad Natl Sci USA 101, 6104–6109 (2004). 10.1073/pnas.0400851101 PMC395930 15079087

[b41] Brown G. C. & Neher J. J. Microglial phagocytosis of live neurons. Nat Rev Neuroscience 15, 209–216 (2014). 24646669 10.1038/nrn3710

[b42] Savill J., Dransfield I., Gregory C. & Haslett C. A blast from the past: clearance of apoptotic cells regulates immune responses. Nat Rev Immunology 2, 965–975 (2002). 12461569 10.1038/nri957

[b43] Aminoff D., Bruegge W. F., Bell W. C., Sarpolis K. & Williams R. Role of sialic acid in survival of erythrocytes in the circulation: interaction of neuraminidase-treated and untreated erythrocytes with spleen and liver at the cellular level. Proc Acad Natl Sci USA 74, 1521–1524 (1977). 10.1073/pnas.74.4.1521 PMC430821 266192

[b44] Jansen A. J. G. . Desialylation accelerates platelet clearance after refrigeration and initiates GPIba metalloproteinase-mediated cleavage in mice. Blood 119, 1263–1273 (2012). 22101895 10.1182/blood-2011-05-355628 PMC3277358

[b45] Samlowski W. W., Spangrude G. J. & Daynes R. A. Studies on the liver sequestration of lymphocytes bearing membrane-associated galactose-terminal glycoconjugates: reversal with agents that effectively compete for the asialoglycoprotein receptor. Cell.Immunol. 88, 309–322 (1984). 6207941 10.1016/0008-8749(84)90164-3

[b46] Vasta G. R. Roles of galectins in infection. Nat Rev Microbiol 7, 424–438 (2009). 19444247 10.1038/nrmicro2146 PMC3759161

[b47] Pangburn M. K., Pangburn K. L., Koistinen V., Meri S. & Sharma A. K. Molecular mechanisms of target recognition in an innate immune system: interactions among factor H, C3b, and target in the alternative pathway of human complement. J.Immunol. 164, 4742–4751 (2000). 10779780 10.4049/jimmunol.164.9.4742

[b48] Lv Y. . Remarkable Activation of the Complement System and Aberrant Neuronal Localization of the Membrane Attack Complex in the Brain Tissues of Scrapie-Infected Rodents. Mol Neurobiol 52, 1165–1179 (2015). 25311207 10.1007/s12035-014-8915-2

[b49] Stevens B. . The classical complement cascade mediates CNS synapse elimination. Cell 131, 1164–1178 (2007). 18083105 10.1016/j.cell.2007.10.036

[b50] Klein M. A. . Complement facilitates early prion pathogenesis. Nat.Med. 7, 488–492 (2001). 11283678 10.1038/86567

[b51] Michel B. . Complement protein C3 exacerbates prion disease in a mouse model of chronic wasting disease. Int Immunol 25, 697–702 (2013). 24038599 10.1093/intimm/dxt034 PMC3900863

[b52] Zabel M. D. . Stromal complement receptor CD21/35 facilitates lymphoid prion colonization and pathogenesis. J Immunol 179, 6144–6152 (2007). 17947689 10.4049/jimmunol.179.9.6144

[b53] Mabbott N. A., Bruce M. E., Botto M., Walport M. J. & Pepys M. B. Temporary depletion of complement component C3 or genetic deficiency of C1q significantly delays onset of scrapie. Nat.Med. 7, 485–487 (2001). 11283677 10.1038/86562

[b54] Kajander T. . Dual interaction of factor H with C3d and glycosaminoglycans in host-nonhost discrimination by complement. Proc Acad Natl Sci USA 108, 2897–2902 (2011). 10.1073/pnas.1017087108 PMC3041134 21285368

[b55] Makarava N. . Genesis of mammalian prions: from non-infectious amyloid fibrils to a transmissible prion disease. PLoS Pathogen 7, e1002419 (2011). 22144901 10.1371/journal.ppat.1002419 PMC3228811

[b56] Savitzky A. & Golay M. J. E. Smoothing and Differentiation of Data by Simplified Least Squares Procedures. Analytical chemistry 36, 1627–1639 (1964).

[b57] Lasch P. & Naumann D. Infrared Spectroscopy in Microbiology. Encyclopedia of Analytical Chemistry, 10.1002/9780470027318.a0117.pub2 (2015).

[b58] Ward J. H. Hierarchical Grouping to optimize an objective function. Journal of American Statistical Association 58, 236–244 (1963).

