# Supplementary material for: Reversible off and on switching of prion infectivity via removing and reinstalling prion sialylation
Source: Sci Rep. 2016 Sep 9;6:33119. doi: 10.1038/srep33119 (PMC5017131; doi:10.1038/srep33119)

Supplementary Information

Reversible off and on switching of prion infectivity via removing and reinstalling prion sialylation

Elizaveta Katorcha[1,2], Martin L. Daus[3], Nuria Gonzalez-Montalban[1,2], Natallia Makarava[1,2], Peter Lasch[3], Michael Beekes[3], Ilia V. Baskakov[1,2#]

[1] Center for Biomedical Engineering and Technology, University of Maryland School of Medicine, Baltimore, Maryland, 21201 United States of America, [2] Department of Anatomy and Neurobiology, University of Maryland School of Medicine, Baltimore, Maryland, 21201 United States of America, [3] Centre for Biological Threats and Special Pathogens, Robert Koch-Institute, 13353 Berlin, Germany

Supplementary Figure 1. (A) Western blot of PK-treated SSLOW brain material serially diluted 10^2-, 10^3-, or 10^4-fold, and PK-treated PMCAb-derived SSLOW material that was not diluted or diluted 10-fold. **(B)** Western blot of non-diluted PMCAb-, dsPMCAb and rsPMCAb-derived SSLOW material. Blots were stained with 3F4 antibody.

Supplementary Figure 1

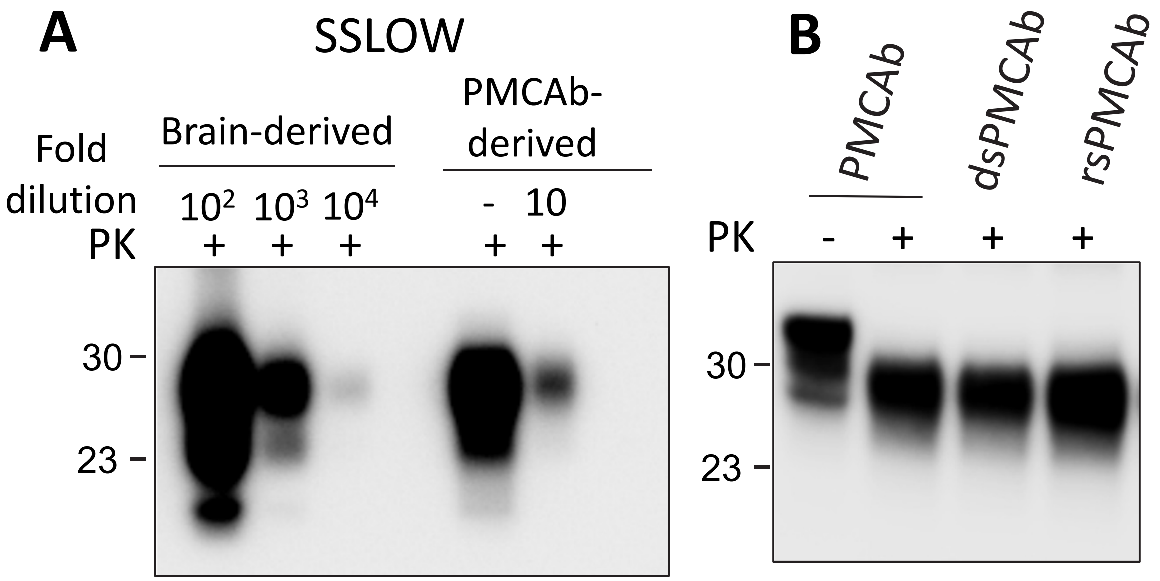

Supplement: Supplementary Information [file srep33119-s1.pdf]
